# Mesenchymal Stem Cells in Early Entry of Breast Cancer into Bone Marrow

**DOI:** 10.1371/journal.pone.0002563

**Published:** 2008-06-25

**Authors:** Kelly E. Corcoran, Katarzyna A. Trzaska, Helen Fernandes, Margarette Bryan, Marcelo Taborga, Venkatesh Srinivas, Kathryn Packman, Prem S. Patel, Pranela Rameshwar

**Affiliations:** 1 Department of Medicine, New Jersey Medical School-UMDNJ, Newark, New Jersey, United States of America; 2 Department of Pathology and Laboratory Medicine, New Jersey Medical School-UMDNJ, Newark, New Jersey, United States of America; 3 Discovery Oncology, Hoffmann-La Roche, Nutley, New Jersey, United States of America; 4 Brookdale University Hospital and Medical Center, Division of Trauma, Brooklyn, New York, United States of America; University of Helsinki, Finland

## Abstract

**Background:**

An understanding of BC cell (BCC) entry into bone marrow (BM) at low tumor burden is limited when compared to highly metastatic events during heavy tumor burden. BCCs can achieve quiescence, without interfering with hematopoiesis. This occurs partly through the generation of gap junctions with BM stroma, located close to the endosteum. These events are partly mediated by the evolutionary conserved gene, *Tac1*.

**Methodogy/Principal Findings:**

This study focuses on the role of mesenchymal stem cells (MSCs), *Tac1*, *SDF-1* and *CXCR4* in BCC entry into BM. The model is established in studies with low numbers of tumor cells, and focuses on cancer cells with low metastatic and invasion potential. This allowed us to recapitulate early event, and to study cancer cells with low invasive potential, even when they are part of larger numbers of highly metastatic cells. A novel migration assay showed a facilitating role of MSCs in BCC migration across BM endothelial cells. siRNA and ectopic expression studies showed a central role for *Tac1* and secondary roles for SDF-1α and CXCR4. We also observed differences in the mechanisms between low invasive and highly metastatic cells. The *in vitro* studies were verified in xenogeneic mouse models that showed a preference for low invasive BCCs to BM, but comparable movement to lung and BM by highly metastatic BCCs. The expressions of *Tac1* and production of SDF-1α were verified in primary BCCs from paired samples of BM aspirates and peripheral blood.

**Conclusions/Significance:**

MSC facilitate BCC entry into BM, partly through Tac1-mediated regulation of SDF-1α and CXCR4. We propose a particular population of BCC with preference for BM could be isolated for characterization. This population might be the subset that enter BM at an early time period, and could be responsible for cancer resurgence and resistance to current therapies.

## Introduction

*Tac1* has been linked to breast cancer (BC) development, and invasion into bone marrow (BM) ([1]–[4]. *Tac1* is ubiquitously expressed, including nervous and hematopoietic systems [5], [6], where its encoded peptides bind to 7-transmembrane, G-protein coupled receptors, neurokinin-1 (NK1), NK2 and NK3 [5]–[8]. The major and most studied *Tac1* peptide is substance P (SP) [9]. Indirect effects of *Tac1* peptides can be partly explained by cytokine production [5], [9].

BC cells (BCCs) express two variants of NK1 with opposing effects on BC development ([2]. *Tac1* is also involved in tumorigenesis through radiation resistance, protection from apoptosis, and induction of growth- and angiogenic-promoting factors [10]. *Tac1* has a central role in BCC entry into BM of nude mice [3]. In BM, when the frequency of BCC is low, *Tac1* mediates the cells' transition to quiescence among stroma, which is located close to the endosteum and also prevent disrupted hematopoiesis [3], [11]. Thus, *Tac1* appears to be central to cancer remission, and also during low tumor burden at an early period, and perhaps prior to clinical detection.

A role for *Tac1* during entry of low invasive and highly metastatic BCCs into BM has not been studied. We report on studies that determined the mechanisms by which mesenchymal stem cells (MSCs) facilitate BCC entry across the blood vessels into BM. MSCs surround the abluminal vasculature of BM and are therefore poised to interface the periphery and BM cavity [12], [13]. Furthermore, MSCs are immune suppressors and could therefore prevent immune clearance of few BCCs, which would be expected during an early period and at low tumor burden [14], [15]. Here we show *Tac1* as a mediator in the coupling of BCCs and MSCs and their migration across BM endothelial cells. The coupling is shown to be partly mediated by double interaction between SDF-1α and its receptor CXCR4 ([16]. SDF-1α belongs to the chemokine family and is ubiquitously expressed [17]. CXCR4 is a seven-transmembrane, G-protein coupled receptor that is involved in chemoattaction of BCCs to organs of high SDF-1α [17]–[21].

## Results

### Interactions between BCCs and MSCs

**Figure 1:**
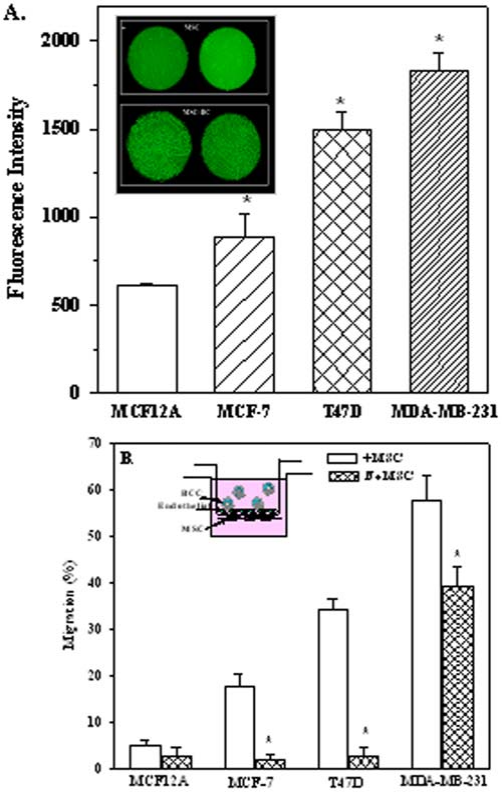
Adhesion and transmigration of BCCs. A. Image is shown of bilayered adherence between MSCs and T47D. B. The adherence of MCF12A, MCF7, T47D and MDA-MB-231 to MSCs are presented as the mean fluorescence intensities, n = 5. Each experiment was done with MSCs from a different donor. C. Migration of BCCs across BM endothelial cells with or without MSCs placed on the lower side of the insert (Refer to inset). The results are presented as the mean percent migration±SD, n = 5. * *p*<0.05 vs. assays with MSCs.

T47D and MCF7 were selected to recapitulate BCC invasion into BM [22], and compared with the highly metastatic, SDF-1^null^ MDA-MB-231 and non-tumorigenic MCF12A [22]. BCCs have been observed to adhere onto MSCs by bilayered method (Figure 1A, inset). A fluorescenc-based quantitative method of adherence showed significantly (*p*>0.05) higher mean fluorescence intensities (±SD, n = 5) for MCF7 and T47D as compared to MCF12A with the highest intensities for MDA-MB-231 (Figure 1A).

### Role of MSCs in Transmigration of BCCs across BM endothelial cells

We established a migration assay to mimic BCC entry into BM across BM endothelial cells and MSCs (Figure 1B, inset). Fluorescence-labeled cells (10^4^) were placed in the inner chamber and transmigrated cells were determined from a standard curve, established with BCCs vs. fluorescence intensities. In the absence of MSCs, the migrations of all BCCs (*p*<0.05) were significantly reduced (Figure 1B). Despite the reduction, MDA-MB-231 showed significant (*p*<0.05) migration in the absence of MSCs as compared to MCF7 and T47D (Figure 1B). In summary, MSCs increased the efficiency of MCF7 and T47D migration through BM endothelial cells, suggesting that the role of MSCs might vary depending on the relevant invasive and metastatic potential of BCCs.

### *SDF-1* and *CXCR4* expression in BCCs

**Figure 2:**
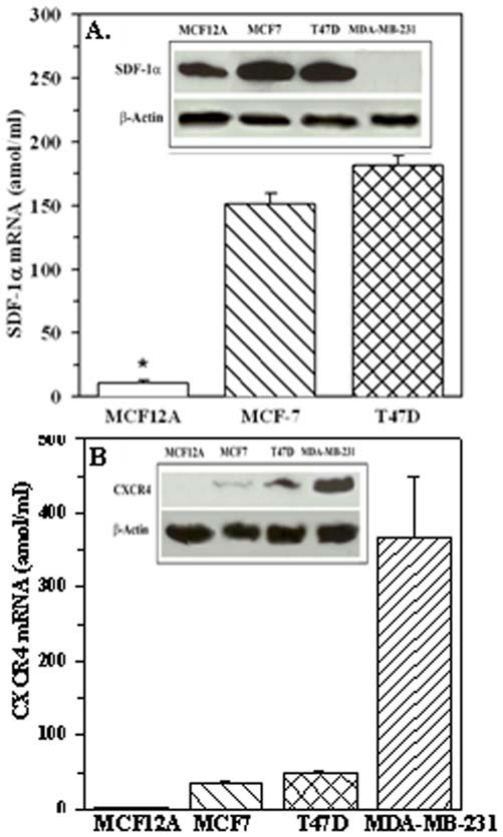
Relative expression levels of CXCR4, and SDF-1α mRNA in BCCs. SDF-1α (A) and CXCR4 (B) mRNA levels were quantitated in BCCs and presented as mean±SD, n = 5. Representative of four western blots for SDF-1α (A, inset) and CXCR4 (B, inset) with membrane extracts from four different cell lines.

SDF-1α and CXCR4 mRNA levels were significantly (*p*<0.05) increased in MCF7 and T47D as compared to MCF12A (Figures 2A and 2B). CXCR4 mRNA was significantly (*p*<0.05) increased in MDA-MB-231 as compared to MCF7 and T47D (Figure 2B). SDF-1α was undetectable, which is consistent with the literature (Figure 2B) [22].

We next examined membrane-bound SDF-1α since this would be relevant for interaction with CXCR4-expressing MSCs [23]. Western blots with membrane extracts showed strong bands for SDF-1α in all cell lines except MDA-MB-231 (Figure 2A, inset). Re-probing for CXCR4 showed light bands for MCF12A and dense bands for the other cell lines (Figure 2B, inset). Normalization with β-actin indicated direct proportion between CXCR4 expression and the cells' aggressiveness, whereas the expressions were similar for MCF7 and T47D.

### Role of CXCR4 on BCC adherence and migration

**Figure 3:**
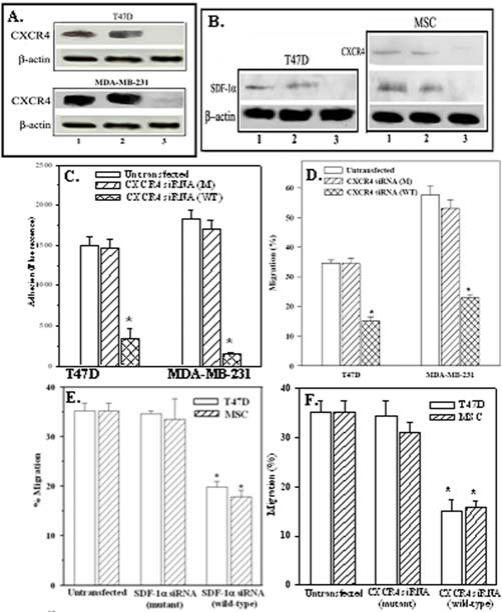
Role of CXCR4 and SDF-1 in BCC migration and adherence. A. Representative of three western blots for CXCR4 and SDF-1α with combined whole cell and membrane extracts from T47D, and CXCR4 for MDA-MB-231. Lanes 1: Untransfected; 2: mutant siRNA; 3: wild-type siRNA. B. Representative of three western blots for SDF-1α (left panel) from three cell passages, and SDF-1α and CXCR4 for MSCs, each from a different donor. C. Adherence studies with T47D and MDA-MB-231 as untransfected, CXCR4 knockdown and control with mutant siRNA, are presented as the mean fluorescence±SD, n = 5. D. Migration studies were done with the same cells and the results presented as mean % migration±SD, n = 5. *p<0.05 vs. untransfected or mutant siRNA or wild-type siRNA. E. Migration studies with T47D, and MSCs knockdown for SDF-1α or mutant siRNA, or untransfected. F. Migration studies were similarly done with CXCR4 knockdown cells. The results are presented as the mean % migration±SD, n = 5. *p<0.05 vs. untransfected or mutant siRNA.

A role for CXCR4 in the adherence and migration of BCCs was studied in cells where CXCR4 was stably knockdown, which was verified by western blots with combined whole cell and membrane extracts (Figure 3A, right lanes). CXCR4 knockdown showed significantly (*p*>0.05) reduced adherence of T47D and MDA-MB-231 to MSCs (Figure 3C) as compared to mutant siRNA and untransfected cells (Figure 3C). Migration studies showed similar observations (Figure 3D). In summary, CXCR4 has roles in the adherence of BCCs to MSCs and migration across BM endothelial-MSCs bilayers.

### SDF-1α-CXCR4 interactions between BCCs and MSCs

We next asked whether double interactions between CXCR4 and SDF-1α are involved in MSC-BCC complexes [24], [25]. To focus, we selected T47D and omitted MDA-MB-231 since they are SDF-1^null^ [26]. SDF-1 or CXCR4 was knockdown in T47D and MSCs and verified gene silencing by western blots (Figures 3A and 3B). There was significant (*p*>0.05) reduction in migration when MSCs or T47D was knockdown for SDF-1 as compared to mutants or untransfected cells (Figure 3E). Similar observations were noted for CXCR4 (Figure 3F). This section showed reduced migration in conditions where SDF-1 or CXCR4 was knockdown in MSCs or T47D.

### Role of *Tac1* on adhesion and migration of BCCs

A critical role has been reported for *Tac1* in BCC entry to BM of nude mice [3]. We now begin to ask whether this could be explained by reduced adherence between BCCs and MSCs. *Tac1* expression was determined by the level of its major peptide, SP ([5]. Its levels (pg/mL±5) were: MCF12A,<5; MCF7, 125±12; T47D, 215±10; MDA-MB-231, 755±20. These values were reduced to <0.02 pg/mL in *Tac1* knockdown cells and was unchanged in untransfected and vector-transfectants.

**Figure 4:**
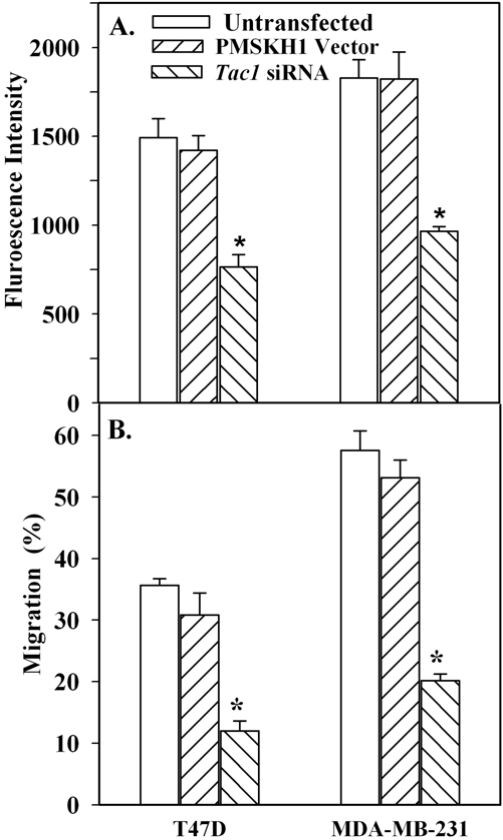
Effects of *Tac1* on adhesion and transmigration of T47D. A. Adhesion of *Tac1* knockdown T47D and MDA-MB-231, vector transfectants or untransfected cells. B. Transmigration studies with the cells described in ‘A’. Results are presented as mean±SD, n = 5. * *p*<0.05 vs. untransfected or vector transfectants.

To focus on further studies with *Tac1* knockdowns, we arbitrarily selected T47D as a low invasive line to compare with MDA-MB-231. Cell adhesion assays showed significant (*p*>0.05) decrease for knockdowns as compared to untransfected and vector transfectants (Figure 4A). Similarly, *Tac1* knockdown showed significant (*p*>0.05) decreases in migration (3-fold) as compared to untransfected and siRNA mutants (Figure 4B). In summary the results show a role for *Tac1* in the adherence and migration of T47D and MDA-MB-231.

### Role of SDF-1α and CXCR4 in the migration of *Tac1* knockdown BCCs

**Figure 5:**
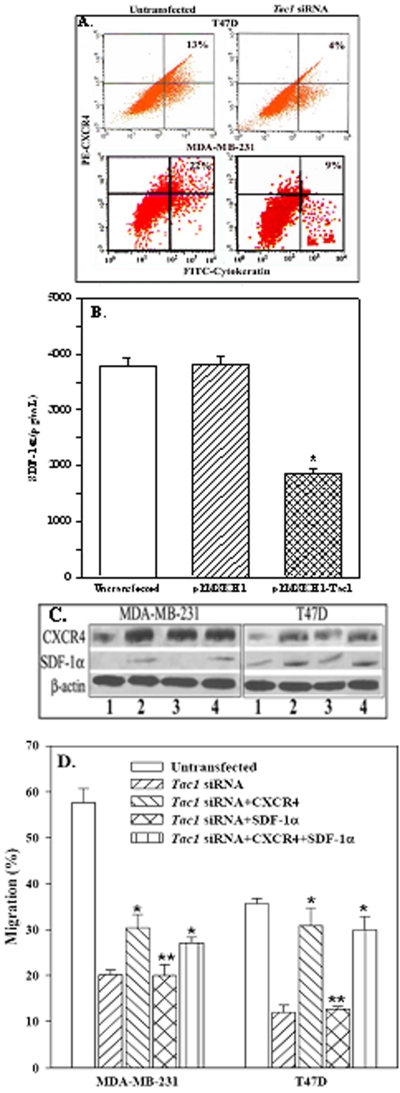
Migration with *Tac1* knockdown T47D and MDA-MB-231, expressed with CXCR4 and/or SDF-1α. A. Cells were analyzed for CXCR4 by flow cytometry (n = 4). B. SDF-1α production by ELISA, mean±SD, n = 5. *p<0.05 vs. untransfected for vector-transfected T47D. C. Western blots with whole cell lysates from *Tac1* knockdown cells and/or expressed for SDF-1α and/or CXCR4 (Lanes 1: *Tac1* siRNA; 2: *Tac1* siRNA+SDF-1α; 3: *Tac1* siRNA+CXCR4; 4: *Tac1* siRNA+SDF-1α+CXCR4. D. Transmigration assays with T47D and MDA-MDB-231 and their variants as for ‘A’. * *p*<0.05 vs. *Tac1* siRNA. ** *p*>0.05 vs. *Tac1* siRNA.

This section determined whether reduced adherence and migration of *Tac1* knockdown BCCs could be explained by changes in SDF-1 and/or CXCR4 expressions. CXCR4 expressions (Figure 5A) were significantly (*p*<0.05) reduced in the *Tac1* cells (Figure 5A). MDA-MB-231^null^ served as negative control. SDF-1α levels over a 24-h period by confluent T47D were significantly (*p*<0.05) reduced in *Tac1* knockdown as compared to vector transfectants and untransfectants (Figure 5B). In summary, *Tac1* knockdown led to reduced SDF-1α production in T47D and lowered CXCR4 expression in both T47D and MDA-MB-231.

SDF-1α and/or CXCR4 were re-expressed in *Tac1* knockdowns to determine if this can over-ride the negative effects of *Tac1* silence with regards to adherence and migration (Figure 5C). CXCR4 expression restored the migration of *Tac1* knockdown T47D, but only partly for MDA-MB-231 (Figure 5D, right diagonal bars), whereas SDF-1α expression showed no difference (Figure 5D, hatched bars). Co-expressions of SDF-1α and CXCR4 in T47D reverted cell migration (Figure 5D, vertical line bars), similar to transfectants with CXCR4 alone (Figure 5D, right hatched bars). The results were specific based on the results with mutant siRNAs (not shown). In summary, CXCR4, but not SDF-1α, restored the migration of *Tac1* knockdown T47D, but only partly for MDA-MB-231.

### *In vivo* verification

This section recapitulates BCC invasion, including an early period of BC. The endpoints are categorized as positive vs. negative, based on the detection of gDNA for β-globin by real-time PCR at 72 h post-injection. β-globin primers did not cross-react with murine gDNA and sensitivity of 1 human cell∶10^6^ murine cells (Figure S1). Time course studies at 24 h, 48 and 72 h for cells in all tissues, peripheral blood, BM, lungs and liver. Although BCCs were detected in BM at 24 h, only the 72-h results were consistent, i.e., each femur within a group was positive. We therefore designate 72 h as the optimum time for studies of BCCs in BM.

The total number of mice positive at the cellular and endosteal regions was significantly (*p*<0.05) reduced in *Tac1* knockdowns (Table 1). The detection of human gDNA was not due to cell fusion between human and murine cells since immunohistochemistry with anti-cytokeratin showed intact BCCs in sections from paraffin-embedded femurs (Text S1, Figure S2). The detection of human gDNA is consistent with the results of PCR with gDNA from peripheral blood (Table 1).

**Table 1:** Effects of Tac1, SDF-1α and CXCR4 in BCC entry into BM.

A. EXPERIMENTAL GROUPS	BM		PB
	Cellular	Endosteal	
**T47D**			
Cell alone	18	18	14
*Tac1* mut	16	18	16
*Tac1* knockdown	1	2	5
- SDF-1α	6	10	6
- CXCR4	16	19	12
- SDF-1α/CXCR4	16	6	14
**MDA-MB-231**			
Cells alone	14	15	15
*Tac1* mut	16	16	16
*Tac1* knockdown	0	1	1
- SDF-1α	14	10	10
- CXCR4	4	4	4
- SDF-1α/CXCR4	10	10	10
Vehicle (PBS)	0	0	0

B. Organs	Time (h)	T47D	MDA-MB-231
Lung	24	0/20	15/20
	48	0/20	18/20
	72	0/20	17/20
Endosteum	24	5/20	5/20
	48	10/20	6/20
	72	18/20	11/20

**BM**: Bone Marrow; **PB**: Peripheral Blood.

**A.** Nude mice (n = 20) were injected with 10^4^ BCCs in the mammary fat pad as untransfected, *Tac1* knockdown, with SDF-1α and/or CXCR4 expressions. At various times, cells from the central region of femurs (Cellular) and those close to the endosteum (Endosteal) were analyzed by real-time PCR for human genomic β-globin (Figure S1). The results are shown for 72 h time points. **B.** Analyses similar ‘A’ were done with gDNA from lungs and endosteal regions of the BM.

We asked if the facilitating role of *Tac1* could be replaced with expressions of SDF-1α and CXCR4. We therefore injected mice with *Tac1* knockdown T47D and MDA-MB-231 that were expressed for SDF-1α and/or CXCR4. While SDF-1α expression showed significantly (*p*<0.05) more positive femurs for MDA-MB-231, CXCR4 expression led to significant (*p*<0.05) increase for T47D (Table 1A). Their co-expressions caused an increase in positive femurs, but not to the level of *Tac1* expression (Table 1A). Also, T47D *Tac1* knockdown cells were less efficient in migrating to the endosteum. Of significance is the detection of MDA-MB-231 after 48 h in lungs whereas T47D was undetectable in lungs even at 72 h (Table 1B). This suggests that T47D shows preference for BM.

We next determined if MSCs are in close location of BCCs in femurs. This was addressed in triple labeled immunohistochemistry for MSCs and BCCs with sections from longitudinal paraffin-embedded femurs of mice injected with T47D for 72 h. Stainings were done for cytokeratin (FITC-green); endothelial cells (CD31-blue) and endothelial/MSCs (CD105-red). Co-labelings for CD31 and CD105 (purple) indicate blood vessel, and/or vessels with surrounding MSCs. Cytokeratin (+) cells in close contact with MSCs (yellow) suggesting close location between MSCs and BCCs. Figure S3, white arrows indicate where BCCs are in close contact with MSCs, based on co-labeling.

**Figure 6:**
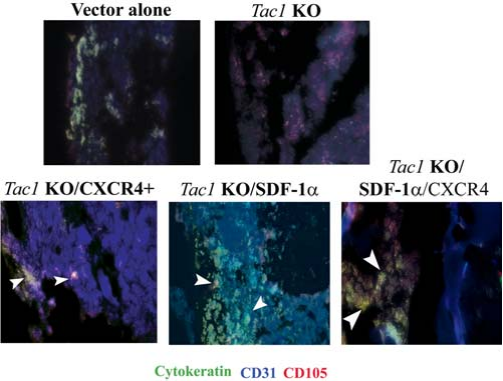
Representative of three sections obtained from femurs nude mice after 24 h of injections with T47D, untransfected, knockdown (KO) for *Tac1*; Tac1 knockdown with re-expressions of SDF-1α and/or CXCR4. The femurs were treated as described for Figure S1 and the slides were triple labeled as for Figure S3 with PE-anti-CD105, FITC-anti cytokeratin and APC-anti-CD31. Arrows in the merged images depict cytokeratin (+) cells in contact with MSCs (green and red), but not in contact with CD31+ cells (blue).

The next set of immunohistochemistry labeled slides at an earlier time point at 24, 48 and 72 h as for Figure S3. The mice were injected with T47D: untransfected, *Tac1* knockdown; *Tac1* knockdown, with SDF-1α and/or CXCR4 re-expressed. Figure 6 shows representative labeling for 24-h injections. The early time point was selected because MSCs are expected to be coupled to BCCs before the cancer cells move towards the endosteum. The slides were triple labeled for cytokeratin (green) and MSCs (red). Since endothelial cells are also positive for CD105, co-labeling for CD31 (blue) served as a marker to discriminate MSCs from endothelial cells. The results are shown for labeling in the cellular region. The images (top panel, left) show yellow labeling for T47D transfected with vector alone, indicating close location between CD105 (MSCs or endothelial cells) and cytokeratin (BCCs) positive cells. Since the yellow image was away from CD31 (blue/endothelial cells), the CD105 represented MSCs. Untransfected T47D showed similar findings (not shown). Cytokeratin cells were not detectable in the *Tac1* knockout cells (top panel, right). Arrows show detectable BCCs in close location with MSCs when SDF-1α and/or CXCR4 were/was re-expressed.

### Substance P-SDF-1α interactions

Re-expression of SDF-1α in the *Tac1* knockdown correlated with enhanced presence of BCCs in the femurs of mice (Table 1). We therefore asked the expressions of *Tac1* and *SDF-1* involves autocrine stimulations. To address this question, we first asked if exogenous SDF-1α (50 ng/mL) enhances the production of substance P in T47D and MDA-MB-231. In addition, we also studied substance P expression in primary BCCs with different stages of BC. Since SDF-1α has been shown to induce the production of substance P in non-tumorigenic MCF12A [27], its stimulation served as control (Table 2). The results show significant increases in substance P for both cell lines and all primary BCCs (Table 2). Interestingly, baseline and induced SDF-1α levels were increased in the late stage disease.

**Table 2:** Induction of substance P by SDF-1α in BC cell lines and primary BC cells.

Cells	Substance P (pg/mL)	
	Unstimulated	Stimulated
MCF12A	0.001±0.0001	71.6±5*
T47D	220±10	565±18*
MDA-MB-231	725±32	1510±25*
P1, P2, P3 (Stage IIIA)	310±22	645±25*
P11, P12 (Stage M0)	162±10	445±18*
P9, P10 (Stage I)	85±5	160±8*

Confluent cells were stimulated for 24 h with 50 ng/mL of SDF-1α in sera-free media. ELISA quantitated the levels of substance P in the culture media. The results are presented as mean substance P levels (pg/mL)±SD. Each cell type was analyzed five times with different cell passages.

* *p*<0.01 vs. unstimulated cells.

The corollary question to determine the effects of *Tac1* expression on SDF-1α production was addressed in knockdown studies. Since MDA-MB-231 is null for SDF-1, the studies were addressed with T47D, knockdown for *Tac1*. SDF-1α levels were significantly (*p*<0.05) decreased in the knockdown cells as compared to untransfected and siRNA mutant (Table 3), indicating that *Tac1* expression is involved in the production of SDF-1α. Since BCCs also express other cytokines [3], we verified that *Tac1* is indeed involved in the production of SDF-1α in a defined model. Thus, we express Tac1 in MCF12A and then studied SDF-1α production during a 24-h period in 80% confluent cells. The results showed significant production of SDF-1α in the expression cells as compared to T47D, untransfected and vector transfectants (Table 4). In summary, the results showed autocrine stimulations by SDF-1α and the major *Tac1* peptide, substance P in T47D.

**Table 3:** SDF-1α production in *Tac1* knockdown T47D.

Cells	SDF-1α (pg/ml)
Untransfected	1108±12
*Tac1* knockdown	105±5*
siRNA vector alone	1215±5
siRNA mutant	1220±15

T47D cells were stably knockdown for *Tac1*. Controls were transfected with mutant siRNA. At 80% confluence, culture media were replaced with fresh media and after aliquots of media were collected and then quantitated for SDF-1α levels by ELISA. The results are presented as the mean±SD of four different experiments.

* *p*<0.05 vs. all other experimental points.

**Table 4:** SDF-1α production in *Tac1*-expressing MCF12A.

MCF12A	SDF-1α (pg/mL)
Untransfected	0.002
Tac1 expression	66±14*
Vector alone	0.001

*Tac1* was ectopically expressed in MCF12A. At 80% confluence, media were replaced. After 24 h, aliquots of media were quantitated for SDF-1α levels by ELISA.

* *p*<0.05 vs. untransfected and vector transfectant.

**Figure 7:**
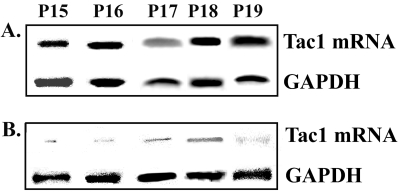
Tac1 mRNA in cytokeratin-expressing cells from BM aspirates and peripheral blood of Stage III BC patients. Pairs of BM aspirates (A) and peripheral blood (B) were taken from BC patients at the time of diagnosis. Cytokeratin positive cells were selected and then subjected to RT-PCR for Tac1 mRNA and GAPDH.

Finally, we determined whether *Tac1* is expressed in BCCs within BM, and also determined how its expression differs from BCCs in the peripheral blood of the same patients. Since the criteria were to get samples at diagnosis, before treatment, we were able to acquire five patients with Stage III BC (P15–P19). RT-PCR for Tac1 mRNA showed bright bands for cytokeratin positive cells in BM aspirates (Figure 7A). Similar studies with cytokeratin expressing cells in peripheral blood showed dim bands (Figure 7B). Despite the small cohort of patients, these results suggest that *Tac1* expression might be enhanced when the BCCs enter BM, or alternatively, *Tac1*-expressing cells might show preference for BM.

## Discussion

This study reports on a BC model that recapitulates an early period when the tumor burden is low, in remission and/or has invaded BM. The model used the low invasive cell lines, MCF7 and T47D and compared with the highly aggressive SDF-1^null^ MDA-MB-231 [22], [26]. The report show a central role for *Tac1*, and a potential facilitating role for MSCs for BCC entry into BM. *Tac1* mediates its effects via SDF-1α and CXCR4. A most interesting finding is the differences observed between the low and highly aggressive BCCs. While *Tac1* is relevant for the biology of both, the involvement of SDF-1α, CXCR4 and MSCs was more relevant for the low invasive lines (Figure 1 and Table 1). Re-expressions of CXCR4 and SDF-1α in *Tac1* knockdown MDA-MB-231 were insufficient to reverse *Tac1* silence (Figures 3 and 5). CXCR4, but not SDF-1α was sufficient to replace the loss of *Tac1* in T47D in migration (Figure 5D). These differences suggest variations at different stages of BC.

The findings underscore relevance for CXCR4 in migration of low metastatic BCCs into BM at low tumor burden, or for a specific population of BCCs during high tumor burden. These differences are important, in particular for the subset that exists during low tumor burden. This population might be responsible for cancer resurgence and could also begin to device translational studies with combinations of available CXCR4 and *Tac1* peptide receptor antagonists. CXCR4 activation depends on the aggressiveness of BCCs [28]. *Tac1* peptides and CXCR4 both activate G-protein coupled receptors [10]. Thus, indepth research studies are required to determine how these receptors are involved in the migration of BCCs through BM endothelial cells, followed by coupling to MSCs, and finally migration towards the endosteum.

The *in vivo* studies have been analyzed by a sensitive method to detect human gDNA in BM and lung (Figure S3 and Table 1). Expression of SDF-1α in *Tac1* knockdown MDA-MB-231 showed no difference in their adherence to MSCs or migration by *in vitro* methods (Figure 5D), but by in vivo studies, SDF-1α showed restoration in the cells' ability to enter BM (Figure 5D, and Table 1A). This observation indicates that other unidentified *in vivo* factors might be involved in BM metastasis and/or invasion by highly metastatic cells such as MDA-MB-231. In contrast, co-expressions of SDF-1α and CXCR4 in *Tac1* knockdown T47D led to an increase in the number of mice with BCCs in femurs, although reversion was not absolute.

An increase in the number of positive femurs with SDF-1α expressing *Tac1* knockdown MDA-MB-231 suggests an advantage for BCCs where SDF-1α production is reduced. Perhaps SDF-1α might occupy CXCR4 through autocrine binding. By occupying CXCR4, the BCCs might lose their efficiency to metastasize towards organs of concentrated SDF-1α.

MDA-MB-231 was detected at an early time point in both BM and lung whereas T47D was only detected in BM (Table 1B). These observations, although preliminary, suggest that the combined *in vitro* and *in vivo* models might be suitable to study the early event of BCC entry into BM, and might also be relevant for the biology of BCCs during low tumor burden without evidence of metastasis.

While CXCR4 is relevant for interaction between BCCs and MSCs, we cannot eliminate the possibility of molecular redundancy and also the involvement of other molecules. *Tac1* knockdown BCCs could still migrate although less, suggesting the involvement of other molecules (Figure 4B). We propose CXCR4 antagonists might be ideal for patients who have been diagnosed early and show no lymph node involvement [29]. This would be a prudent measure to prevent low numbers of BCCs to enter BM.

Previous studies have reported BCCs with varying cycling properties, based on their location in BM [3]. In this study, we have subjected the femurs to strong wash before the cells were scraped, indicating strong attachment of BCCs to the endosteal compartment where hematopoietic stem cells are located. While information is beginning to emerge on the mechanisms by which BCCs are able to retain hematopoietic homeostasis through gap junctions and changes in SDF-1α levels [11], robust analyses are required to determine how various stages of the hematopoietic hierarchy are affected by the two major subsets of BCCs, and also to characterize them at the molecular and phenotypic levels. In addition, further studies are required to understand how gap junctions affect the properties of BCCs with BM cells and bone [11], [30], [31].

Roles for MSCs during an early period of BC need consideration, especially since others have supported BM aspirates for early diagnosis [32]. MSCs surround the abluminal surface of the blood vasculature of BM could act against the immune system and protect BCCs in BM [12], [13], [32]. If the entering BCCs have relatively few mutations, this population is likely to be protected by MSCs [33]–[36]. We emphasize that the proposed role for MSCs at an early period and/or at low tumor burden is different from metastasis [37].

In contrast to roles for CXCR4 in BC metastasis [26], this study focuses on its role during an early period, including a role during entry into BM and the potential involvement of MSCs at the interface between the BM and periphery. Although the *in vitro* studies show a facilitating role for MSCs in the migration of BCCs, a definitive role for MSCs is still to be determined. The *in vivo* studies show close locations between BCCs and MSCs. Since depletion of MSCs could lead to overt vascular damage, it is difficult to show a role for MSCs. An understanding of relevant molecules in the interactions between BCCs and MSCs will allow for future studies to have definitive answers for the role of MSCs in BCC survival and quiescence in BM. This report has underscored future studies to track the movement of BCCs in live animal using imaging with luciferase expressing cells.

The study has raised questions on drug combinations with CXCR4 and Tac1 receptor antagonists for both low and highly metastatic BCCs. Figure S4 summarizes the findings and show BCCs entering BM and interacting with MSCs through double interactions between SDF-1α and CXCR4. Upon entry, all or few BCCs reach the endosteum to form gap junctions with BM stroma [11]. While we have studied SDF-1α, other isoforms might be involved [37]. An understanding of the mechanisms by which BCC enter and survive in BM could lead to pertinent treatments, detection, and methods to ‘flush’ BCCs from BM for eradication. An understanding of BCC survival in BM is significant since BC could resurge after ten years of remission, and is associated with poor prognosis [38], [39].

## Materials and Methods

### Mice

Female athymic BALB/c mice (4 weeks) were obtained from the National Cancer Institute (Bethesda, MD) and housed in a laminar flow hood at an AALAC-accredited facility. The use of mice was approved by the Institutional Animal Care and Use Committee, New Jersey Medical School (Newark, NJ). BCCs (10^4^) were injected into the left, hind mammary fat pad, and then euthanized at 48 and 72 h post-injection. BM cells designated as those from cellular region were obtained by slowly flushing femurs with media through a syringe, attached to a 26 g needle. Cells designated as those in the endosteal regions were obtained by opening femurs longitudinally and then scraping the cells attached to the inner surfaces.

### Cells

The following cell lines were purchased from American Type Culture Collection (www.atcc.org): MCF-7, T47D, P815, MCF12A and MDA-MB-231. MSCs were cultured from human BM aspirates in DMEM with 10% FCS (D10 media), as described [14]. The use of human BM aspirates followed a protocol approved by the Institutional Review Board (IRB-UMDNJ, Newark campus). The MSCs were adherent cells, morphologically symmetric, CD14^−^, CD29^+^, CD44^+^, CD34^−^, CD45^−^, SH2^+^, and negative for prolyl-4-hydroxylase [15].

### Primary BCCs

The following samples were obtained from five different patients with Stage III BC (P15–P19) at Brookdale Hospital, Brooklyn, NY and at University Hospital, University of Medicine and Dentistry of New Jersey (UMDNJ), Newark, NJ: breast tissues from surgical specimens; peripheral blood cells and BM aspirates. The samples were left-over from diagnostic procedures, before treatment. The use of tissues was approved by the institutional review board of Brookdale Hospital and UMDNJ. The samples were obtained before the patients were placed on treatments with anti-cancer agents. The demographics of Patients 15–19 (P15–P19) were as follows: Age ranges between 45 and 62 yrs; P15–P18 were positive for estrogen (ER) and progesterone (PR) receptors and Herceptin (HER2) negative. P19 was negative for ER and PR and HER (+). Surgical tissues and peripheral blood samples were obtained from P15–17. Peripheral blood and BM aspirates were taken from P18 and P19.

The hormone status and age of Stage IIIA patients 1–3 (P1–P3); Stage M0, P11 and P12 and Stage 1, P9 and P10 were previously reported [2], [3]. The malignant cells from these patients were expanded from surgical tissues, as described [40].

### BM Endothelial Cells (BMECs)

BMECs were cultured from BM aspirates of healthy individuals, as outlined by a protocol approved by the IRB-UMDNJ. Mononuclear cells were separated by Ficoll-Hypaque density gradient and then plated in fresh endothelial medium (Cambrex, Boston MA). Cells were incubated until confluence (∼3 weeks). Media (50%) were replaced with fresh lot at weekly intervals. Flow cytometry indicated that >95% at passage 5 were positive for vWF and CD31.

### Vectors

pPMSKH1/*Tac1* was previously described [3]. pPMSKH1-SDF-1/KC, an SDF-1-specific siRNA vector was constructed as previously described [3]. Mutants contained three single base pair changes: pPMSKH1-SDF-1/KM. pSUPER-CXCR4 (wild-type and mutant) siRNA vectors were kindly provided by Dr. Si-Yi Chen (Baylor University) [41]. CXCR4 expression vector was kindly provided by Dr. Nelson Michael (Walter Reed Research Institute) [42]. pEF2-SDF-1α expression vector contained the coding region of Acc#L36034. pEF2 was provided by Dr. Sergei Kotenko (UMDNJ) [43].

### Stable Expressions

BCCs or MSCs were co-transfected with pTK-Hyg and pPMSKH1-*Tac1*, pPMSKH1-SDF-1/KC, pPMSKH1-SDF-1/KM, pSUPER-CXCR4 (mutant and wild-type), or pPMSKH1. Transfectants were selected with hygromycin or G418. *Tac1* knockdown showed undetectable SP by ELISA [3]. *CXCR4* knockdown showed no evidence of membrane and intracellular expressions by western blots. SDF-1 knockdown was confirmed by negative RT-PCR.

*Tac1* knockdown BCCs were stably transfected with CXCR-4 and/or SDF-1α expression vectors as described above. SDF-1α expression was verified by ELISA and western blots as above, and CXCR4 by flow cytometry and western blots with membrane extracts. Flow cytometry studies were done with cells de-adhered with Dissociation Solution (Sigma).

### CXCR4 and SDF-1α mRNA Levels

CXCR-4 and SDF-1α mRNA levels were determined with Quantikine mRNA kit (R&D Systems) using 5 µg of total RNA, as per manufacturer's instructions. Unknowns were calculated with standards provided with the kit. The assay limits were 5 amol/mL for both SDF-1α and CXCR-4.

### Western Analyses

Membrane extracts were prepared with 2×10^6^ cells as described [2], and 15 µg were analyzed by western blots on gradient SDS-PAGE (BioRad). Proteins were transferred onto polyvinyl membranes and then incubated first with primary antibodies, 1/1000 final dilutions and then with secondary antibodies at 1/2000 dilutions. Bands were detected by chemiluminescence.

### SP ELISA

ELISA quantitated SP as described [2]. At confluence, media were replaced with 2% FCS-containing media. After 24 h, media were collected and then quantitated by ELISA. Unknowns were analyzed as undiluted and two serial dilutions, each studied as triplicates. The unknowns were determined with a standard curve established for each plate.

### Cell Adhesion Assay

Adhesion of BCCs to MSCs was studied with the Cell Adhesion Assay Kit (Invitrogen, Carlsbad, CA). MSCs (10^4^/well) were incubated overnight in 96-well plates. BCCs were labeled with the fluorescent cytoplasmic tracer, Vybrant CFDA SE (Invitrogen) as per manufacturer's instructions and 10^3^ were added to the confluent MSC. Non-specific binding was studied in wells without MSCs. After 15 min, the non-adherent cells were washed twice with PBS and the adherent cells were detected by fluorescence on the FL1500 Fluorescent Microplate Reader (Biotek, Winooski, VT). Non-specific adherence was subtracted from the test wells.

### Transmigration Assay

BCC migration used a Boyden chamber with 8 micron inserts. The inner wells were inverted and 10^3^ MSCs were added to the filter in D10 media. The next day, the wells were overturned and then placed into the outer chamber contained 500 µL of D10 media. BMECs (10^4^) were added to the inner chamber in endothelial media. The next day, BCCs were labeled with CellTracker™ Green CMFDA (5-chloromethylfluorescein diacetate) (Invitrogen) as per manufacturer's instruction. Briefly, 10^6^ BCCs were incubated for 15 min with 5 µM of CMFDA. Labeling efficiency by fluorescence microscopy indicated >95% labeling efficiency. BCCs (10^4^) were added to the inner chamber in sera-free DMEM. After 3 h, cells were washed twice with PBS. The cells in the inner chamber were removed with a cotton swab and 500 µl of PBS were added to the outer chamber. The inserts were examined for fluorescence on the Typhoon (Amersham Pharmacia, Molecular Dynamics, Sunnyvale, CA). The mean fluorescence intensity for each well was quantitated using Image Quant software and the percent cell migration was calculated on a standard curve of total BCCs vs. fluorescence intensity.

### Real-time PCR

gDNA was isolated from T47D (human) at log_10_ fold dilution ranging from 10^4^ to 1, added to 10^6^ or 2×10^6^ P815 (murine). gDNA quality was tested by standard PCR at 55°C for 40 cycles in a GeneAmp PCR 9700 thermal cycler (Applied Biosystems, Foster City, CA), Platinum Taq polymerase (Invitrogen) and murine IL-10 primers, Acc#M37897, +1523/+1723. Real-time PCR for human gDNA was done with primers for β-globin or growth hormone with Roche Light Cycler 2.0. Gene specific primers and probes were purchased as part of the Control Kit DNA for Light Cycler. Each sample was analyzed in triplicate and the data analyzed by Poisson's law of small numbers in which one, two or three positives were counted positive for human cells. PCR at 40 cycles/55°C T_M_ showed consistent detection at 1 T47D∶10^6^ P815 with β-globin primers, but inconsistent results with growth hormone primers. Thus, all analyses shown for the *in vivo* studies were done with β-globin primers.

### Semi-quantitaive RT-PCR

Cytokeratin expressing cells were isolated from BM aspirates and peripheral blood of patients (P15–P19) as described [44]. Total RNA was isolated with the RNAqueous 4PCR kit (Ambion, Austin, TX) and then subjected to reverse-transcription with SuperScript III Reverse Transcriptase (Invitrogen). The cDNA served as template to amplify Tac1 mRNA. PCR was done with Platinum Taq Polymerase (Invitrogen) under the following conditions: 95°C, 30 sec; 55°C, 30 sec; and 72°C, 30 sec for 35 cycles. The reaction was preceded by an initial denaturation at 95°C for 2 min and a final extension at 72°C for 10 min. PCR was normalized by amplifying the same cDNA with primers for GAPDH under similar PCR conditions. Tac1 primers span +60 to +328 (NM_003182), 5′-act gtc cgt cgc aaa atc-3′ (sense) and 5′-ggg cca ctt gtt ttt caa-3′ (antisense). GAPDH primers span +254 to +851 (NM_002046), 5′-cca ccc atg gca aat tcc atg gca-3′ (sense) and 5′-tct aga cgg cag gtc agg tcc acc-3′ (antisense). All PCR reactions were analyzed by electrophoresis on a 1% agarose gel containing ethidium bromide and fragment sizes were compared with 1 kb plus DNA ladder (Invitrogen).

### Statistical Analyses

Statistical evaluations were done with analysis of variance and Tukey-Kramer multiple comparisons test. *p*<0.05 was considered significant. For small numbers, the data were analyzed by Poisson law of small numbers, and by binomial probability distribution [45].

## Supporting Information

Text S1 (0.03 MB DOC)

Figure S1 The sensitivity of detecting human gDNA was studied with different ratios of T47D cells (human) to P815 (murine). Representative graph shows the sensitivity of 1 T47D among 106 P815 (arrow).
(0.07 MB PDF)

Figure S2 Representative section from ten femurs of nude mice, injected with T47D or MDA-MB-231. The femurs were sectioned after 72 h of injection and then embedded as longitudinal sections. A. Control comprised section of breast tissue from a patient with Stage III BC, labeled with FITC-isotype control (right panel) or FITC-anti-cytokeratin (left panel). B. Section from a femur injected with T47D (left panel) or MDA-MB-231 (right panel).
(0.09 MB PDF)

Figure S3 Representative of five sections obtained from femurs nude mice, injected with T47D. The femurs were treated as described for Figure S1 and the slides were triple labeled with PE-anti-CD105, FITC-anti cytokeratin and APC-anti-CD31. The latter was done by indirect staining with APC-anti-mouse IgG. Each primary and the secondary antibody were used at 1/2000 final dilution. MERGED figure shows arrows depicting cytokeratin (+) cells in contact with CD105+/CD31- cells.
(0.09 MB PDF)

Figure S4 Shown in cartoon, are untransfected BCCs, Tac1 knockdown BCCs, with SDF-1α or CXCR4 expressed, entering the BM cavity, in complex with MSCs. While the untransfected BCCs have been shown to form gap junctions with stromal cells close to the endosteum (1). The fate of the other two BCCs to reach the stromal compartment has not been shown, and is currently unclear.
(0.28 MB PDF)
