# Supplementary material for: Mesenchymal Stem Cells in Early Entry of Breast Cancer into Bone Marrow
Source: PLoS One. 2008 Jun 25;3(6):e2563. doi: 10.1371/journal.pone.0002563 (PMC2430536; doi:10.1371/journal.pone.0002563)
Supplement: Text S1 — (0.03 MB DOC) [file pone.0002563.s001.doc]

**Supplemental Information**

# Title: Mesenchymal stem cells in early entry of breast cancer into bone marrow.

**Methods:**

Reagents:

β-actin mAb was purchased from Sigma (St. Louis, MO); SDF-1α and rabbit anti-SDF-1 from R&D Systems (Minneapolis, MN); rabbit anti-CXCR4 from Affinity Bioreagents (Golden, CO); HRP-goat anti-rabbit and goat anti-mouse IgG from Santa Cruz Biotechnology (Santa Cruz, CA); PE-anti-CD105 from RDI Division of Fitzgerald Industries Intl (Concord MA); APC-rabbit anti-mouse IgG from Open Biosystems (Huntsville, AL). The following antibodies were purchased from BD Pharmingen (San Jose, CA): PE- and FITC-IgG isotype, PE-CXCR4 mAb; FITC-cytokeratin mAb. The following were obtained from Dako (Carpinteria, CA): CD31, vWF, and prolyl-4-hydroxylase mAbs.

Immunohistochemistry of mice femurs:

Mice were euthanized and both femurs were snipped at each end. One femur was cut longitudinally and the other served as a source of cells for qPCR. The femurs were immediately fixed in 4% paraformaldehyde (PFA) for at least 7 days, and then decalcified for 24 h in decalcifying solutions (Polysciences Inc., Warrington, PA). After this, the femur was embedded in Paraplast Plus (Thermo Electron Co., Waltham, MA). Tissues were sectioned at 6 micron slices and then mounted onto positively charged microscope superfrost glass slides (Fisher Scientific, Springfield, NJ). The slides were exposed to two xylene washes, each for 10 min. After this, the slides were sequentially exposed to 100%, 95%, 70% and 50% ethanol baths, 2 min each. Slides were then washed for 1 min in distilled water and then transferred to a 95^o^C, 10 mM citrate buffer bath (pH 6) for 30 min. After this, the slides were slowly cooled to room temperature. The specimens were treated with Na borate for 10 min at room temperature, and then washed thrice with Tris buffered saline for 5 min.

The next step subjected the slides to immunofluorescence for cytokeratin in the case of Figure 1S. Slides were incubated for 30 min with normal mouse serum at 1/10,000, diluted in tris buffered saline and 0.15% H_2_O_2_. After this, slides were incubated for 1 h with 0.5µg/ml of FITC-mouse anti-cytokeratin IgG (Becton Dickinson) or 0.5 µg/ml of FITC-isotype control (Open Biosystems,). The sections were rinsed with distilled water three times and the slides were immediately examined with an inverted immunofluorescence microscope (Nikon Eclipse TS100).

In triple labeling studies, tissues were labeled first by indirect staining with primary anti-CD31 for 2 h followed by 1-h incubation with secondary APC-rabbit anti-mouse IgG. After this, slides were washed five times and then labeled with combinations of PE-anti CD105 and FITC-anti cytokeratin for 2 h.
