# Supplementary figures and images for: Mesenchymal Stem Cells in Early Entry of Breast Cancer into Bone Marrow
Source: PLoS One. 2008 Jun 25;3(6):e2563. doi: 10.1371/journal.pone.0002563 (PMC2430536; doi:10.1371/journal.pone.0002563)

**Figure S1**

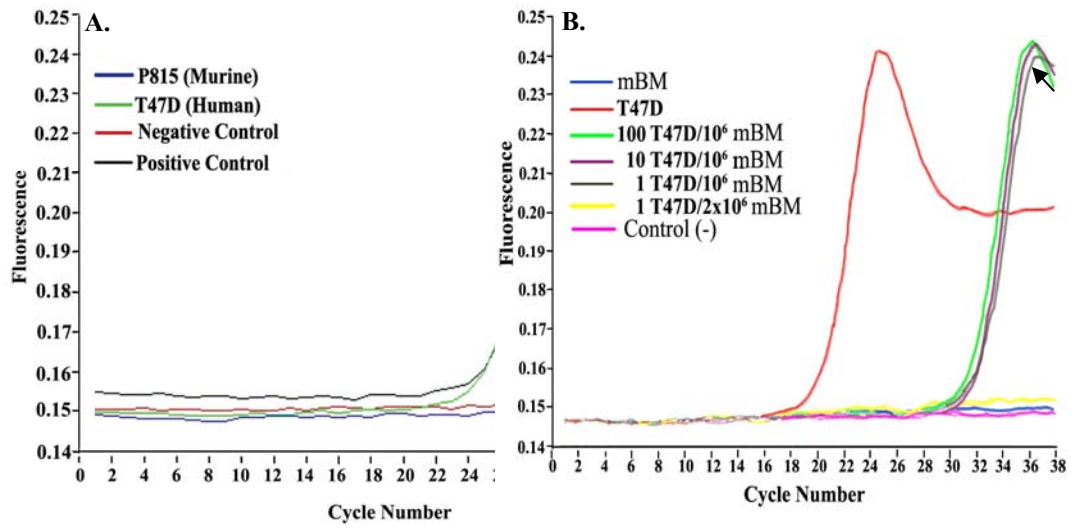

Supplement: Figure S1 — The sensitivity of detecting human gDNA was studied with different ratios of T47D cells (human) to P815 (murine). Representative graph shows the sensitivity of 1 T47D among 106 P815 (arrow). (0.07 MB PDF) [file pone.0002563.s002.pdf]

**Figure S2**

**Positive Control**

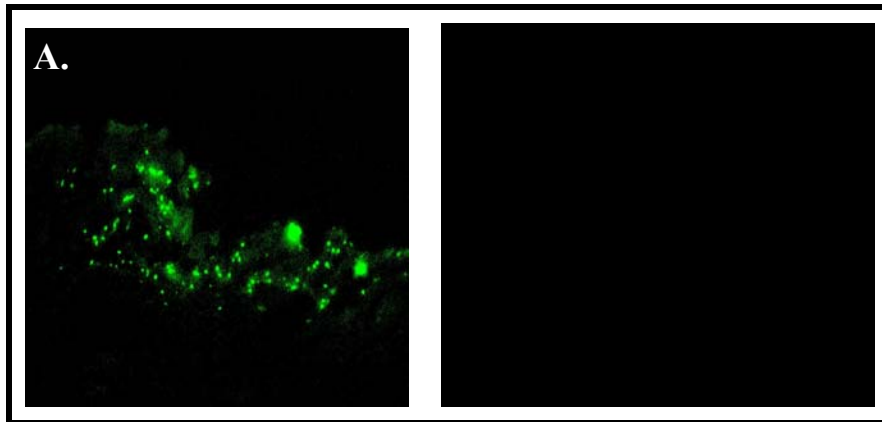

**T47D**

**MDA-MB-231**

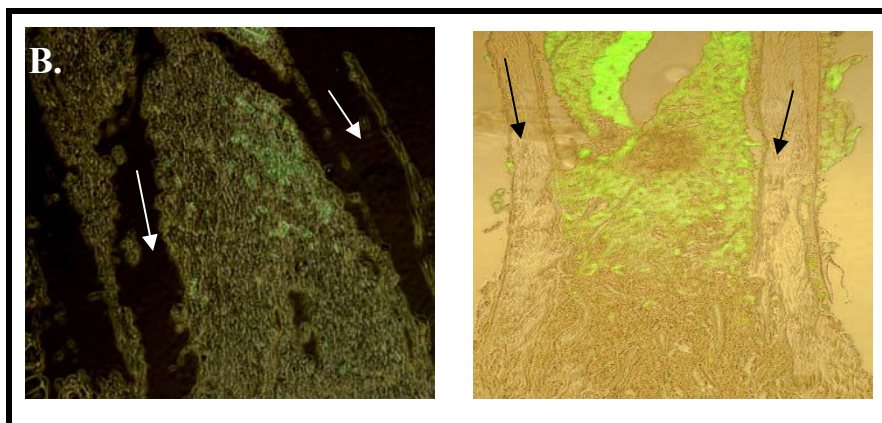

Supplement: Figure S2 — Representative section from ten femurs of nude mice, injected with T47D or MDA-MB-231. The femurs were sectioned after 72 h of injection and then embedded as longitudinal sections. A. Control comprised section of breast tissue from a patient with Stage III BC, labeled with FITC-isotype control (right panel) or FITC-anti-cytokeratin (left panel). B. Section from a femur injected with T47D (left panel) or MDA-MB-231 (right panel). (0.09 MB PDF) [file pone.0002563.s003.pdf]

Figure S3

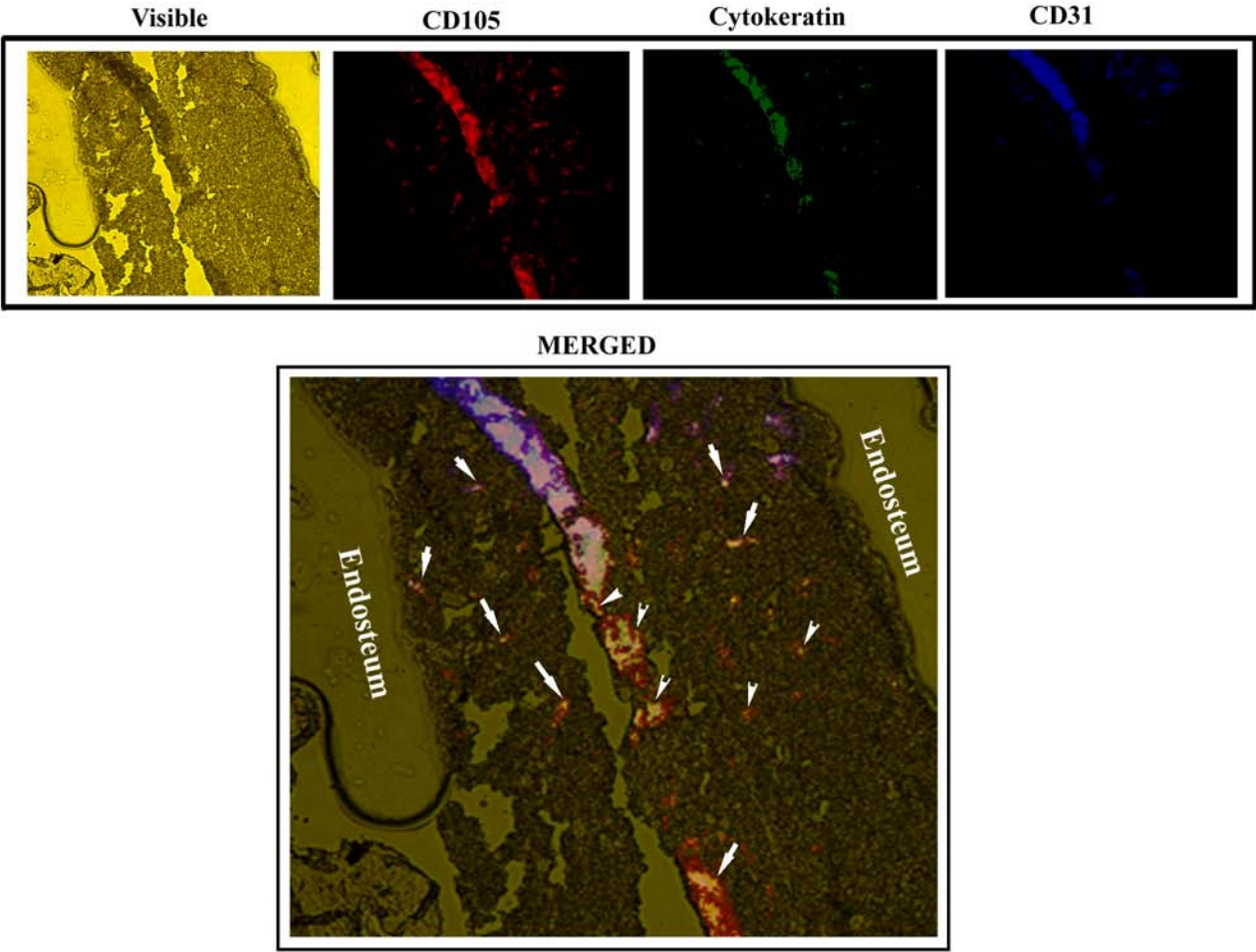

Supplement: Figure S3 — Representative of five sections obtained from femurs nude mice, injected with T47D. The femurs were treated as described for Figure S1 and the slides were triple labeled with PE-anti-CD105, FITC-anti cytokeratin and APC-anti-CD31. The latter was done by indirect staining with APC-anti-mouse IgG. Each primary and the secondary antibody were used at 1/2000 final dilution. MERGED figure shows arrows depicting cytokeratin (+) cells in contact with CD105+/CD31- cells. (0.09 MB PDF) [file pone.0002563.s004.pdf]

Figure S4

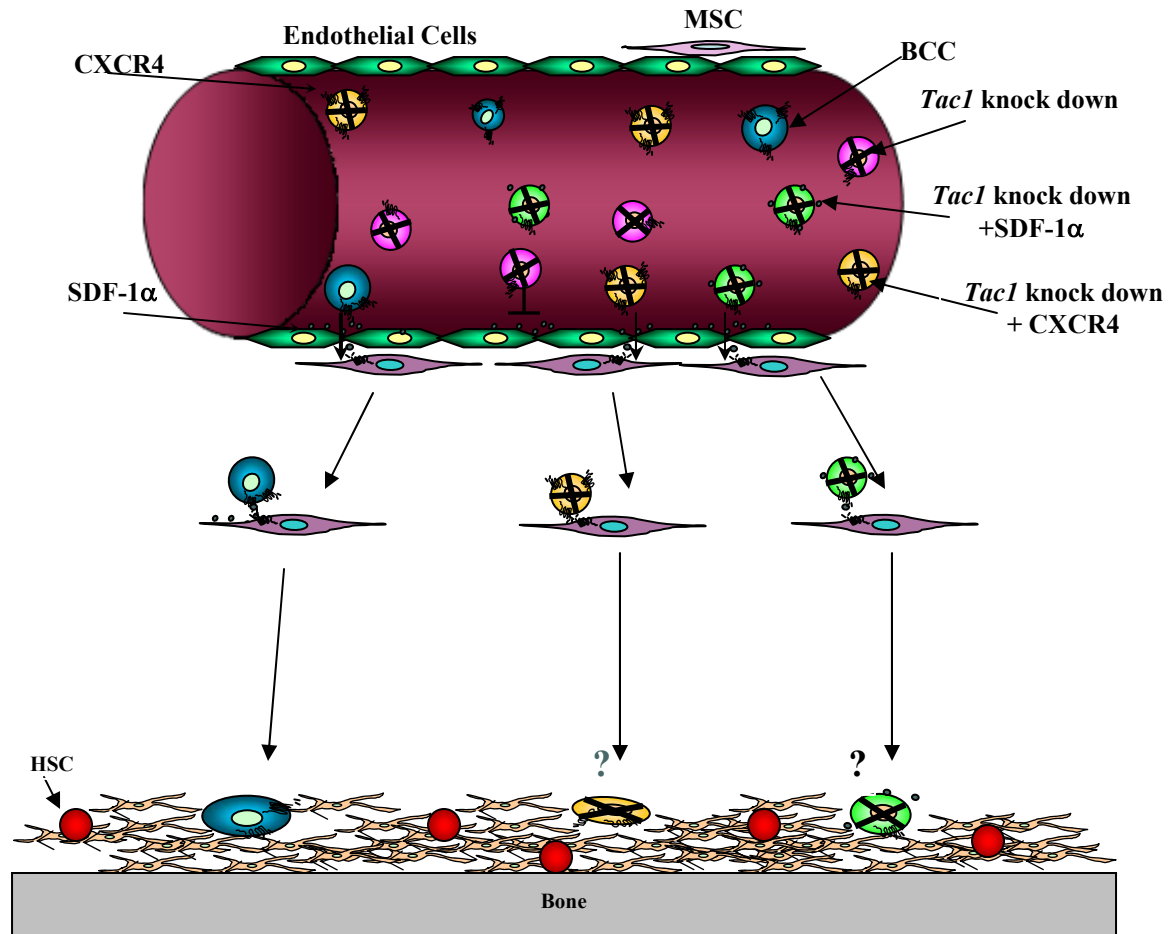

Supplement: Figure S4 — Shown in cartoon, are untransfected BCCs, Tac1 knockdown BCCs, with SDF-1α or CXCR4 expressed, entering the BM cavity, in complex with MSCs. While the untransfected BCCs have been shown to form gap junctions with stromal cells close to the endosteum (1). The fate of the other two BCCs to reach the stromal compartment has not been shown, and is currently unclear. (0.28 MB PDF) [file pone.0002563.s005.pdf]
